# Thiamine deficiency unrelated to alcohol consumption in high‐income countries: a literature review

**DOI:** 10.1111/nyas.14569

**Published:** 2021-02-11

**Authors:** Filomena Gomes, Gilles Bergeron, Megan W. Bourassa, Philip R. Fischer

**Affiliations:** ^1^ The New York Academy of Sciences New York New York; ^2^ Pediatric and Adolescent Medicine Mayo Clinic Rochester Minnesota; ^3^ Department of Pediatrics Sheikh Shakhbout Medical City Abu Dhabi United Arab Emirates

**Keywords:** thiamine deficiency, nonalcoholic, case reports, case studies

## Abstract

Thiamine deficiency has been typically associated with alcoholism or as a prevalent problem in low‐ and middle‐income countries (LMICs) whose populations rely on staple foods with a low content of thiamine. We conducted a literature review of published and unpublished data to identify relevant adult cases with confirmed thiamine deficiency of nonalcoholic cause in developed countries. We selected 17 reports with 81 adult cases of confirmed thiamine deficiency affecting adult patients with a wide range of ages and underlying conditions (e.g., cancer, gastrointestinal diseases, heart failure, and obesity). Thiamine deficiency may have been caused by disease‐related malnutrition, bariatric surgery, chronic use of diuretics, repeated vomiting, lack of thiamine in parenteral nutrition formulas, food insecurity, and reliance on monotonous or restrictive diets. Treatment with intravenous thiamine resulted in partial or complete recovery from the symptoms (cardiac, neurologic, and metabolic disorders) for most patients. The number and variety of symptomatic thiamine‐deficient adults identified in this review demonstrates that thiamine deficiency is not exclusive to LMICs and, in high‐income settings, is not exclusive to alcoholic patients. In developed countries, this serious but treatable condition can be expected in patients suffering from various medical conditions or following certain dietary patterns.

## Introduction

Thiamine (a B‐complex vitamin) plays a key role in energy metabolism and in the proper functioning of the multiple organ systems, such as the nervous, musculoskeletal, and cardiovascular systems.^1^, ^2^ Thiamine deficiency has been increasingly recognized as an important problem in low‐ and middle‐income countries (LMICs). In these settings, populations that rely on staple crops with low thiamine content (such as polished rice or cassava), consume antithiamine factors (such as betel nuts and fermented fish), or follow postpartum restrictive diets have suffered from thiamine deficiency disorders. The pediatric population is known to be particularly susceptible to thiamine deficiency. Infantile beriberi mostly affects exclusively breastfed infants whose mothers are thiamine deficient, and it results in death within hours of clinical presentation if not promptly treated.^1^ All of these facts are discussed in other articles of this special issue.^3^, ^4^

While food fortification with thiamine is less common in LMICs, in some high‐income countries, fortified foods (e.g., breads and breakfast cereals) contribute to about half of the total amount of thiamine consumed.^5^ In highly resourced countries, thiamine deficiency is considered to be very rare because of food fortification and access to a varied diet, and is most commonly associated with alcoholism.^6^ The excessive chronic consumption of alcohol is a well‐known cause of thiamine deficiency and the consequent Wernicke encephalopathy (due to impaired intestinal absorption and utilization of thiamine); thus, when facing a patient with a history of alcoholism, clinicians tend to look for symptoms of thiamine deficiency.^7^, ^8^ However, in the absence of a medical history of alcoholism, the variable symptoms of thiamine deficiency (which affects multiple organ systems) may be attributed to other conditions and can be easily misdiagnosed.^2^, ^6^, ^9^ With this literature review, we aim to provide an overview of adult cases of confirmed thiamine deficiency of nonalcoholic origin that have been reported in high‐income countries. In particular, we aim to capture the variety of clinical manifestations of thiamine deficiency disorders.

## Materials and methods

Using the electronic bibliographic database Medline, via PubMed, we conducted literature searches to identify relevant reports of adult cases with confirmed thiamine deficiency of nonalcoholic cause in high‐income countries. We used the search terms “thiamine deficiency” or “beriberi,” in combination with one or more of the following keywords: “adult,” “report,” “case report,” “case series,” “nonalcoholic,” “polyneuropathy,” “encephalopathy,” “neuropathy,” and “metabolic/lactic acidosis.” In addition to searching published material, we also identified abstracts from conferences and unpublished data. We considered confirmation of thiamine deficiency as per the judgment of the authors of each report/case study, which were based on improvement of symptoms after the administration of thiamine, and/or on laboratory assessment of thiamine blood levels, and/or on clinical history/assessment (e.g., neuroimaging findings). Cases reported in the pediatric population, in adults with a current history of excessive alcohol consumption, or from LMICs were excluded. The definition of countries with high‐income economies was based on the most recent World Bank classification.^10^ We described the overall characteristics of each identified case, the likely cause of thiamine deficiency, how it was treated, whether treatment resulted in the resolution of symptoms, the identified delays in diagnosis (when reported), and potential associations with infections.

## Results

We included 17 selected relevant reports with 81 adult cases (or clusters of cases) of confirmed thiamine deficiency of nonalcoholic origin that were observed in high‐income countries (Table 1). These cases were described in published papers, conference abstracts, and in a personal communication (i.e., shared via email, within our network of thiamine experts). Most reports (nine) were identified in the United States; three were observed in Japan, one in Italy, one in France, one in the United Kingdom, one in Korea, and one in Saudi Arabia, between 1999 and 2020. The 81 patients included in this analysis were between 19 and 75 years old and 69% were males. Most of the cases (94%) had an underlying health condition (such as cancer, gallstone, pancreatitis, heart failure, or a recent surgery or medical treatment that caused weight loss); a smaller number of cases (6%) did not present with a background disease but experienced some level of food insecurity, followed a particular dietary pattern according to personal preferences (e.g., ate mostly white rice and disliked meat and vegetables), or ate a restrictive diet to encourage weight loss. Thus, there was a wide range of likely causes of thiamine deficiency, including disease‐related malnutrition (usually accompanied by a significant, unintentional weight loss), gastrointestinal symptoms and surgical complications following bariatric surgery, food insecurity, chronic use of diuretics (to manage heart failure), repeated vomiting, lack of thiamine in parenteral nutrition formulas, and reliance on monotonous or restrictive diets. Body mass indexes, when reported, varied from underweight (13 kg/m^2^) to obese (43 kg/m^2^). Blood thiamine levels were measured in the majority of the patients (94%), with most presenting low levels compared with the laboratory reference ranges. The symptoms and clinical presentation varied significantly from case to case, with a wide range of neurologic and cardiac manifestations typical of thiamine deficiency (e.g., muscle weakness, peripheral neuropathy, confusion, edema, tachycardia, dyspnea, and metabolic acidosis), in agreement with the existing literature. The initial treatment of thiamine deficiency in the various cases was mostly intravenous, and when reported, the dose varied from 75 mg once daily to 500 mg three times a day. Despite this variability in the dose of thiamine administered, treatment resulted in partial or complete recovery from the symptoms for most of the patients, except in three cases that resulted in death.^11^, ^12^ Infections were present in 11 cases: one with a septic state,^13^ one with complex intra‐abdominal infections,^14^ and nine that did not specify the type of infection.^15^

**Table 1:** Nonalcoholic, adult cases of thiamine deficiency reported in high‐income countries

Source	Country, year(s) of data collection	Number of cases and institution or type of setting (e.g., hospital and community)	Overall characteristics: age, gender, and underlying condition(s)	Likely cause of thiamine deficiency	Laboratory confirmation	Symptoms and clinical presentation	Treatment: supplementation dose and effect on symptoms (resolution)	Other notes (including the presence or absence of infection)
Alligier *et al*.^12^ (Published article) “A series of severe neurologic complications after bariatric surgery in France: the NEUROBAR Study”	France, 2010–2018	38 cases with neurologic complications after bariatric surgery, of which 14 had confirmed thiamine deficiency	34 females, 4 males; median age = 39 years; median BMI = 43 kg/m^2^; 34% received gastric bypass and 45% sleeve gastrectomy, with neurologic complications observed 6 months (median) after surgery	Gastrointestinal symptoms and surgical complications after bariatric surgery (e.g., vomiting and limited oral energy intake)	Thiamine deficiency was confirmed in 14 cases (values not provided)	10 cases had encephalopathy, 15 had peripheral neuropathy, 12 had both, and 1 had a pyramidal syndrome	15 patients received IV, and 2 received oral thiamine supplementation; neurologic symptoms were completely resolved in 9 cases; 2 patients died	No reported infection
Kohnke and Meek^18^ (Published article) “Don't seek, don't find: the diagnostic challenge of Wernicke's encephalopathy”	United Kingdom, 2020	One case admitted to hospital (department of surgery)	26‐year‐old woman had bariatric surgery 6 weeks before	Persistent vomiting for 6 weeks after gastric sleeve surgery	Not known (“inconclusive biochemistry” assessed after onset of treatment)	Presented with nystagmus, imbalance, and gait disturbance, interfering with activities of daily living	250 mg IV thiamine (3×/day), later raised to 500 mg (3×/day) for several weeks, resulting in a marked improvement of symptoms	No reported infection
Zafar^30^ (Published article) “Wernicke's encephalopathy following Roux‐en‐Y gastric bypass surgery”	Saudi Arabia, 2015	One case admitted to hospital	40‐year‐old male had Roux‐en‐Y gastric bypass surgery (for weight loss) 3 months before and reported repeated vomiting since then	Repeated vomiting (and likely little oral food intake) following weight loss surgery	No	Presented with confusion and difficulty in maintaining balance while walking; assessment: nystagmus and ataxic gait (Wernicke encephalopathy)	500 mg IV thiamine (3×/day), then 250 mg/day, resulted in improved symptoms (“almost completely normal” on 3‐month follow‐up visit)	No reported infection
Isenberg‐Grzeda *et al*.^15^ (Published article) “Nonalcoholic Thiamine‐Related Encephalopathy (Wernicke–Korsakoff Syndrome) Among Inpatients With Cancer: A Series of 18 Cases”	U.S., 2013–2014	18 cancer inpatients with Wernicke–Korsakoff syndrome referred to psychiatry service	Median age = 65 years; 33% women; 61% with solid tumors and 39% with hematologic malignancies	Disease‐related malnutrition: decreased availability/intake (e.g., nausea), accelerated usage (e.g., infection and fever), impaired use (e.g., fluorouracil and metronidazole), and excessive loss of thiamine	Yes, in 89% of patients: serum thiamine was <7 nmol/L (normal: 9–14 nmol/L)	Presented with cognitive signs and symptoms; for example, altered mental status (100%), cerebellar signs and symptoms (39%), and ocular signs and symptoms (17%). No/little vitamins B_9_ and B_12_ deficiency	Most received 500 mg IV thiamine (3×/day), initiated on average 18 days after symptom onset; 17% had complete resolution of symptoms, and 83% had residual symptoms at the time of last follow‐up	50% of patients had an infection
Cui and Qiu^2^ (Published article) “Thiamine Deficiency (Beriberi) Induced Polyneuropathy and Cardiomyopathy: Case Report and Review of the Literature”	U.S., 2014	One case admitted to the ER	20‐year‐old female with papillary thyroid carcinoma and dysphagia caused by radiation injury; low body weight	Significantly decreased oral intake due to dysphagia	Yes, thiamine was 7 nmol/L (normal 9–14 nmol/L)	Presented with bilateral lower extremity weakness and paresthesia, inability to walk, chest palpitations, and shortness of breath; assessment: ptosis, nystagmus, tachycardia, and lactic acidosis	IV thiamine supplementation for 5 days and tube feeding after hospital discharge resulted in the improvement of all symptoms at 3‐month follow‐up visit	No reported infection
Jung *et al*.^11^ (Published article) “Wernicke's Encephalopathy in Advanced Gastric Cancer”	Korea, 2009	Two cases admitted to hospital	Case 1: 48‐year‐old woman with advanced GC, receiving intermittent home parenteral nutrition (HPN), suffered a 20‐kg weight loss over 2 months Case 2: 58‐year‐old woman with advanced GC and a 15‐kg weight loss over 2 months	Case 1: disease‐related malnutrition (with marked weight loss after chemotherapy) Case 2: disease‐related malnutrition (with marked weight loss after chemotherapy)	Case 1: No Case 2: No	Case 1: presented with dizziness and diplopia; assessment: nystagmus and gaze disturbance (Wernicke encephalopathy) Case 2: presented with sudden disorientation, confusion; assessment: gaze limitation and mild ataxia (Wernicke encephalopathy)	Case 1: daily parenteral injection of thiamine 100 mg for 17 days resulted in improved symptoms Case 2: parenteral injection of thiamine (100 mg for 4 days), but patient had recurrent seizure attack and aggravation, resulting in death on hospital day 6	Case 1: no reported infection Case 2: no reported infection. Thiamine replacement started 3 days after neurologic symptoms and was ineffective
Helali *et al*.^31^ (Published article) “Thiamine and Heart Failure: Challenging Cases of Modern‐Day Cardiac Beriberi”	U.S., 2018	Two cases admitted to the ER	Case 1: 68‐year‐old homeless obese man (BMI = 33 kg/m^2^) Case 2: 63‐year‐old obese man (BMI = 39 kg/m^2^) was severely limiting caloric intake to encourage weight loss	Case 1: food insecurity Case 2: restrictive diet (less than 1 meal/day, mostly convenience foods)	Case 1: Yes, a random nonfasting level of 12 nmol/L, a few days after admission (normal: 8–30 nmol/L) Case 2: Yes, undetectable	Case 1: presented with progressive dyspnea and swollen legs; assessment: cardiomegaly and anemia; multiple hospital visits in the following 3 months, with new neurocognitive deficits and bilateral cranial nerve 6 palsies Case 2: presented with dyspnea and altered mental status; assessment: tachycardia, tachypnea, and severe heart failure	Case 1: 100 mg oral thiamine/day improved cardiac and cognitive function after 16 days Case 2: heart failure improved significantly after 14 days of IV thiamine	Both cases: no reported infection. Authors suggest that patients who present with an unexplained cardiomyopathy should be evaluated for thiamine deficiency
Misumida *et al*.^32^ (Published article) “Shoshin Beriberi Induced by Long‐Term Administration of Diuretics: A Case Report”	U.S., 2014	One case admitted to the ER; Transferred to ICU on second day	61‐year‐old man with a history of heart failure (receiving furosemide and trichlormethiazide therapy for 6 months), diabetes and stage 3 chronic kidney disease. BMI = 29 kg/m^2^	Chronic diuretic therapy	Yes, plasma thiamine concentration of 11 mg/dL (normal range: 20–50 mg/dL)	Presented with dyspnea; assessment: edema in legs, cardiomegaly, pulmonary vascular congestion, and severe metabolic acidosis	IV thiamine supplementation (100 mg/day) resolved all symptoms and patient was discharged on day 15 (on “oral vitamin pills”)	No reported infection (absence of fever and leukocytosis, and negative results of serial blood cultures)
Romanski and McMahon^33^ (Published article) “Metabolic Acidosis and Thiamine Deficiency”	U.S., 1999	One case admitted to medical center	19‐year‐old woman, BMI = 13 kg/m^2^, with persistent, unexplained GI symptoms and receiving HPN	Absence of multivitamins, most significantly thiamine, in HPN formula (no multivitamins or trace elements were provided for 19 days)	No, test was ordered but not completed; reason not provided	Presented with nausea, vomiting, diarrhea, and abdominal pain; assessment: very low BMI, hyperglycemia, and metabolic acidosis	IV thiamine supplementation (100 mg daily for 2 days), followed by daily administration of 50 mg orally for the next 14 weeks, resulted in a dramatic clinical improvement	No reported infection
Koike *et al*.^34^ (Published article) “Myopathy in thiamine deficiency: analysis of a case”	Japan, 2006	One case admitted to hospital	26‐year‐old woman with a particular dietary pattern (ate mostly white rice and drank coffee, disliked meat and vegetables); BMI = 24.5 kg/m^2^	Monotonous diet	Yes, total thiamine in whole blood was 16 ng/mL (normal, 20–50 ng/mL)	Presented with walking difficulties, leg edema, and myalgia; assessment: moderate cardiomegaly with pulmonary congestion and axonal neuropathy	75 mg oral dose of fursultiamine (daily) resulted in dramatic decrease in cardiomegaly, pleural effusions, and edema in the legs, followed by improvement of neurologic symptoms, muscle strength, and myalgia	No reported infection
Shible *et al*.^13^ (Published article) “Dry Beriberi Due to Thiamine Deficiency Associated with Peripheral Neuropathy and Wernicke's Encephalopathy Mimicking Guillain–Barré syndrome: A Case Report and Review of the Literature”	U.S., 2019	One case admitted to hospital and transferred to intensive care unit	56‐year‐old woman with history of gallstone pancreatitis and malnutrition; on HPN until 6 months prior to admission, then returned to normal diet. BMI unknown	Underlying severe protein‐calorie malnutrition and duration of critical illness	Yes, but measured after four doses of thiamine therapy (serum level: 104 nmol/L, reference range: 70–180 nmol/L)	Presented with paresthesia of the lower limbs, arms and neck; assessment: unresponsive to verbal stimuli, Hb 9.4 g/dL, and Wernicke encephalopathy	High‐dose IV thiamine (500 mg every 8 h) resulted in mental status improvement within 48 hours	Reported infection (septic state). Initial diagnosis was Guillain–Barré syndrome (symptoms and signs of dry beriberi can mimic those of the Guillain–Barré syndrome)
Koike *et al*.^17^ (Published article) “Rapidly developing weakness mimicking Guillain–Barré syndrome in beriberi neuropathy: two case reports”	Japan, 2007	Two cases admitted to hospital	Case 1: 46‐year‐old man had a gastrectomy 3 years ago to treat cancer Case 2: 33‐year‐old man with a particular dietary pattern (did not like meat or vegetables, preferring white rice and noodles with no side dishes); heavy outdoor work	Case 1: disease‐related malnutrition (BMI or weight loss not reported) Case 2: monotonous diet	Case 1: Yes, total thiamine in whole blood was 15 ng/mL (normal: 20–50 ng/mL) Case 2: Yes, total thiamine in whole blood was 7 ng/mL (normal: 20–50 ng/mL)	Case 1: presented with weakness in lower extremities; assessment: axonal neuropathy; later developed progressive weakness, lactic acidosis, and heart failure Case 2: presented with weakness of limbs (unable to walk); assessment: severe sensory deficits in legs and mild sensory loss in hands	Case 1: 100 mg IV thiamine resulted in gradual improvement of all symptoms Case 2: 100 mg IV thiamine resulted in gradual improvement of all symptoms	Both cases: no reported infection and symptoms mimicked Guillain–Barré syndrome (which was initially considered as a diagnosis)
Solorzano and Guha^14^ (Published article) “Wernicke's Encephalopathy: Under Our Radar More Than it Should Be?”	U.S., 2016	One case admitted to hospital	30‐year‐old woman with abdominal sepsis due to choledocholithiasis, on parenteral nutrition due to poor oral intake (caused by nausea, vomiting, and abdominal pain)	Thiamine was not included in the parenteral nutrition formulation	Yes, low levels (values not provided)	Presented with deteriorated mental status; assessment: miotic pupils and roving eye movements, and Wernicke encephalopathy	500 mg IV thiamine, 3×/day for 3 days, then oral 50 mg/day resulted in symptom improvement, although ataxia and memory issues persisted 2 months later	Reported complex intra‐abdominal infections
Ruiz *et al*.^35^ (Conference abstract) “Acute polyneuropathy and Wernicke encephalopathy due to thiamine deficiency”	Italy, 2019	One case admitted to the ER	59‐year‐old woman with cancer (adenocarcinoma of the extrahepatic biliary tree)	Disease‐related malnutrition	Yes, low serum thiamine level (34 nmol/L, normal values 66–200)	Admission: subacute onset of confusion, amnesia for recent events and confabulation. Later: nystagmus, hypoesthesia, and severe flaccid quadriparesis	200 mg of IV thiamine hydrochloride/day, for 3 weeks Strength improvement, distal lower limb paresthesia, reduced reflexes at upper limbs and areflexia at lower limbs. Amnestic‐confabulatory syndrome persisted	No reported infection
Murase *et al*.^36^ (Conference abstract) “Shoshin beriberi in a young man living on Japanese rice balls”	Japan, year not reported	One case admitted to ER	24‐year‐old single man, living alone, no underlying conditions (BMI = 17.4 kg/m^2^)	Food insecurity (subsisting on balls of polished rice in preceding 4 years) due to financial problems	Yes, 17 ng/mL (normal: 24–66 ng/mL)	Presented with chest pain and shortness of breath; assessment: systemic edema, central cyanosis, hyporeflexia, lactic acidosis, and moderate cardiomegaly	Hemodynamic parameters improved dramatically in only 3 h after thiamine administration (dose not reported)	No reported infection
Bruera *et al*.^37^ (Clinical communication to the editor) “The Malnourished Heart: An Unusual Case of Heart Failure”	U.S., 2017	One case admitted to hospital	66‐year‐old woman with no past medical history, but relied on a diet “of processed cheese chips and vanilla cake” for 2 years; BMI = 17 kg/m^2^	Monotonous diet that led to multiple nutritional deficiencies	Yes, low levels (values not provided)	Presented with worsening dyspnea, lower extremity edema, and orthopnea; assessment: heart failure, periodontal disease, patchy hair loss, low HB (10.3 g/dL), deficient for vitamins B1, B6, C, and D	After injections of thiamine and other vitamins + multivitamin tablets, the heart failure symptoms resolved completely and periodontal disease improved	No reported infection
Mates (2020) (Personal communication, unpublished data*^a^* from Dr. Elisabeth Mates, MD, attending hospitalist physician at the VA Sierra Nevada Healthcare System)	USA, 2018–2020	33 cases admitted to hospital	Veteran patients (mean age = 75 years old) from the greater Reno area, Nevada, with wide range of underlying acute and chronic illnesses	Disease‐related malnutrition (illnesses or conditions that led to reduced appetite and nutritional intake, e.g., cancer, cholecystitis, and pancreatitis)	Yes, all patients had a plasma thiamine level ≤7 nmol/L (normal: 8–30 nmol/L); note: takes 7–10 days to obtain test result	Wide‐ranging symptoms; no single symptom stands out other than many had weakness and “hospital delirium”	Treated patients (<50%) had demonstrable improvement in the neurologic symptoms and general weakness; some went from needing nursing home level of care to be discharged home after treatment	Pending subsequent prospective study (*n* = 400) to determine the prevalence of thiamine deficiency in hospitalized patients

BMI, body mass index; ER, emergency room; GC, gastric cancer; GI, gastrointestinal; HPN, home parenteral nutrition; ICU, intensive care unit; IV, intravenous; WB, whole blood.

a Manuscript in preparation for peer review; data included in this table reflect the information provided by Elisabeth Mates, MD, an attending hospitalist physician at the VA Sierra Nevada Healthcare System.

## Discussion

The present work provides an overview of the variety of conditions or dietary modifications that can lead to thiamine deficiency and are not linked to excessive chronic consumption of alcohol. It demonstrates that thiamine deficiency is not a resolved problem and is not exclusive to LMICs; it is still present even in areas where it is not expected. It highlights that, in high‐income countries, thiamine deficiency affects many more patients beyond the expected cases caused by alcoholism. These may include patients with cancer, heart failure, gastrointestinal diseases, or surgeries in the gastrointestinal tract that lead to reduced intake and absorption of nutrients (e.g., gastrectomy to treat cancer or gastric bypass to promote weight loss). Other causes of nonalcoholic thiamine deficiency include the inappropriate formulation of parenteral nutrition (even when used for only a few weeks) and the chronic use of diuretics, which are used to manage fluid and sodium levels in heart failure (among other health conditions) but can cause hyperexcretion of thiamine.^16^ Thus, the clinical community should consider thiamine deficiency in these patient populations that present with cardiac and neurologic symptoms, as well as metabolic acidosis and muscular symptoms. The pathophysiologic mechanisms that can lead to thiamine deficiency are summarized in Table 2.

**Table 2:** Pathophysiologic mechanisms that can lead to thiamine deficiency in adults

Pathophysiologic mechanisms	Causes	References
Increased thiamine requirements	Malignancy	11, 15
	Fever and infection/sepsis	23
	Refeeding syndrome	38
	High‐carbohydrate diets	25
Increased thiamine losses	Hemodialysis and peritoneal dialysis	39, 40
	Chronic diuretic therapy	32
	Prolonged vomiting	18, 29
	Prolonged diarrhea	41
Decreased thiamine intake or absorption	Alcoholism	7
	Bariatric surgery	12
	Malnutrition	13
	Restrictive or poor quality diet	36, 37
	Parenteral nutrition (inappropriate formulation)	33, 42
	Hyperemesis gravidarum	29
	Foods containing thiamine antagonists and thiaminases	43, 44

Healthcare professionals are relatively unaware of thiamine deficiency as a possible cause of polyneuropathy, particularly in patients without Wernicke encephalopathy or heart failure in the initial phase, and some of the thiamine deficiency cases we identified were initially diagnosed as Guillain–Barré syndrome.^13^, ^17^ Even in patients with Wernicke encephalopathy, it is estimated that 80% of the cases do not receive a diagnosis, with some cases only being diagnosed postmortem.^18^ A delayed diagnosis of thiamine deficiency is a serious problem because, if intravenous thiamine is not administered soon after the onset of symptoms, the patient's clinical condition can deteriorate very quickly and even result in death.^17^, ^19^ Early symptoms of thiamine deficiency, such as nausea, vomiting, fatigue, weakness, and milder neurological manifestations, are often not attributed to thiamine deficiency by clinicians.^20^

It usually takes a few days to obtain the results of a laboratory assessment of blood thiamine levels. Some authors recommend obtaining a thiamine level before thiamine replacement (to help confirm the diagnosis), without delaying the treatment, that is, beginning the thiamine administration immediately after the blood is collected for analysis.^2^, ^14^ Thiamine has no tolerable upper intake level (there is no established toxic level) and is safe and inexpensive, justifying the prompt treatment. It is also recommended that the thiamine infusion should precede or be given along with intravenous glucose, as glucose (alone) can precipitate Wernicke encephalopathy in thiamine‐deficient individuals due to increased utilization of thiamine.^14^, ^21^, ^22^

Infections can increase the use of thiamine and precipitate Wernicke encephalopathy in patients with a suboptimal thiamine status; in addition, infections have been shown to lead to worse neuropsychological outcomes in thiamine‐deficient patients.^23^ In the present study, severe infections were reported in only two cases, and another nine cases did not specify the type of infection, although it should be noted that infections may have been present in other patients but not reported in the description of the case study.

Body mass index, when reported in the case study, varied from 13 to 43 kg/m^2^, showing that thiamine deficiency can affect underweight, normal weight, overweight, and even obese patients. The history of a significant unintentional weight loss (which can be as high as 20 kg in 2 months^11^) is, however, a sign that the patient is malnourished or at risk of malnutrition, even in the presence of abundant fat reserves. When unintentional weight loss is coupled with the presence of neurological or cardiac symptoms, it should alert the clinician to the suspicion of thiamine deficiency. This literature review revealed a significant number of obese individuals who became thiamine deficient after undergoing weight‐loss surgery—a phenomenon that has been called “bariatric beriberi.”^24^ Bariatric surgery can lead to thiamine deficiency because postoperative vomiting is common and because the area of the gut available for nutrient absorption is reduced in certain procedures (e.g., Roux‐en‐Y gastric bypass), resulting in decreased thiamine absorption. Patients with a history of bariatric surgery are, therefore, recommended to take daily thiamine supplements indefinitely.^18^ In addition, thiamine deficiency seems to affect a significant proportion (15–29%) of preoperative bariatric surgery patients. This suggests that obese patients are a group at risk of thiamine deficiency, which may be explained by the typical dietary pattern of high consumption of simple sugars (which contain very little thiamine and require relatively high amounts of this vitamin for their metabolism) and low consumption of foods high in thiamine (such as whole grains, legumes, and seeds).^25^

Other previously published studies have looked at specific manifestations of thiamine deficiency, that is, Wernicke–Korsakoff syndrome not related to alcohol use,^26^ Wernicke encephalopathy in schizophrenia,^27^ Wernicke encephalopathy in patients with depression,^27^ Wernicke encephalopathy following gastrointestinal surgeries,^28^ Wernicke encephalopathy in hyperemesis gravidarum,^29^ and Wernicke encephalopathy after bariatric surgery.^24^ The present work, despite not being an exhaustive nor a comprehensive list of cases found in the literature, includes a variety of cardiac, neurologic, muscular, and metabolic manifestations of thiamine deficiency not associated with excessive alcohol consumption. This is a major strength of our review and an approach that, to our knowledge, has not been used before. For example, the thiamine‐deficient oncologic patients identified in our literature review presented with a wide range of clinical manifestations, from Wernicke encephalopathy^11^ and Wernicke–Korsakoff syndrome^15^ to polyneuropathy and cardiomyopathy.^2^ In these cancer patients, thiamine deficiency can be caused by several mechanisms, including decreased thiamine availability/intake (e.g., low oral intake, low appetite, nausea, and oral thrush), accelerated usage (e.g., the presence of infection and fever), impaired functioning (e.g., use of medications, including metronidazole and fluorouracil, which inactivate thiamine), and excessive loss (e.g., due to vomiting and diarrhea).^15^ In summary, our work suggests that thiamine deficiency associated with certain underlying diseases can have diverse clinical manifestations.

Hopefully, this article will raise awareness about the need for early recognition and appropriate treatment of thiamine deficiency in a variety of conditions not associated with alcoholism. While thiamine deficiency can result in serious consequences, prompt treatment can lead to a full recovery of the affected patient.

## Author contributions

F.G. performed the literature searches, extracted data, and drafted the manuscript. All authors were involved in the conception, design, revision of the manuscript, and the approval of its final version.

## Competing interests

The authors declare no competing interests.
